# Maximal information component analysis: a novel non-linear network analysis method

**DOI:** 10.3389/fgene.2013.00028

**Published:** 2013-03-12

**Authors:** Christoph D. Rau, Nicholas Wisniewski, Luz D. Orozco, Brian Bennett, James Weiss, Aldons J. Lusis

**Affiliations:** ^1^Division of Cardiology, Department of Medicine, David Geffen School of Medicine, University of CaliforniaLos Angeles, CA, USA; ^2^Department of Microbiology, Immunology and Molecular Genetics, University of CaliforniaLos Angeles, CA, USA; ^3^Department of Human Genetics, David Geffen School of Medicine, University of CaliforniaLos Angeles, CA, USA; ^4^Department of Physiology, David Geffen School of Medicine, University of CaliforniaLos Angeles, CA, USA

**Keywords:** gene expression, ICMg, scale-free topology, MINE, GxE interactions

## Abstract

**Background:** Network construction and analysis algorithms provide scientists with the ability to sift through high-throughput biological outputs, such as transcription microarrays, for small groups of genes (modules) that are relevant for further research. Most of these algorithms ignore the important role of non-linear interactions in the data, and the ability for genes to operate in multiple functional groups at once, despite clear evidence for both of these phenomena in observed biological systems.

**Results:** We have created a novel co-expression network analysis algorithm that incorporates both of these principles by combining the information-theoretic association measure of the maximal information coefficient (MIC) with an Interaction Component Model. We evaluate the performance of this approach on two datasets collected from a large panel of mice, one from macrophages and the other from liver by comparing the two measures based on a measure of module entropy, Gene Ontology (GO) enrichment, and scale-free topology (SFT) fit. Our algorithm outperforms a widely used co-expression analysis method, weighted gene co-expression network analysis (WGCNA), in the macrophage data, while returning comparable results in the liver dataset when using these criteria. We demonstrate that the macrophage data has more non-linear interactions than the liver dataset, which may explain the increased performance of our method, termed Maximal Information Component Analysis (MICA) in that case.

**Conclusions:** In making our network algorithm more accurately reflect known biological principles, we are able to generate modules with improved relevance, particularly in networks with confounding factors such as gene by environment interactions.

## Introduction

High throughput biological technologies, such as transcriptome microarrays, have enabled researchers to query biological networks that underlie cellular processes and pathways involved in diseases. Examination of these pathways has led to the discovery of novel biological targets (Gargalovic et al., 2006; Horvath et al., 2006; Dewey et al., 2011; Park et al., 2011). A common form of biological network is the co-expression network, constructed by analyzing the pairwise relationships between RNA transcripts across a set of perturbations (Stuart et al., 2003; Zhang and Horvath, 2005; Keller et al., 2008; Langfelder and Horvath, 2008; Barabási et al., 2011; Park et al., 2011). In these networks, genes whose expression patterns are related to one another form the links or edges of the graph, while the genes themselves form the nodes or vertices. A common means of analyzing co-expression networks relies on algorithms that partition the network into clusters or modules, consisting of genes having strong associations with each other. These modules assist researchers in the identification of key genes and interactions in a biological process by dramatically reducing the overall complexity of the data from thousands of individual genes to a small number of functional components.

**Figure 1:**
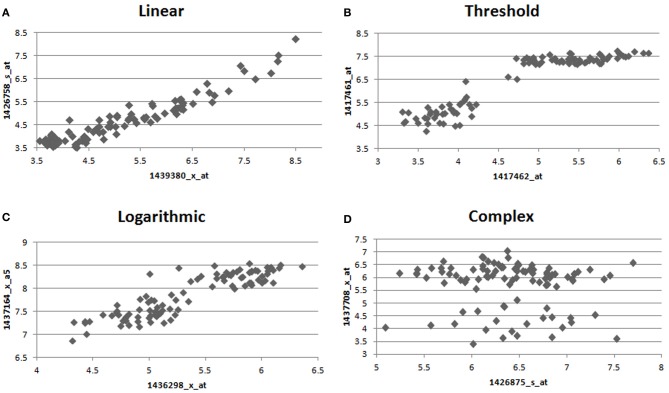
**Example relationships observed between pairs of genes in the macrophage dataset.** We observe many different forms of relationships, such as **(A)** linear **(B)** high threshold **(C)** logarithmic **(D)** complex, where different relationships are observed between treated and control macrophages. Only interactions of type **(A)** are preferred by Pearson correlation, while all interactions are treated equally by MIC.

Many computational methods (Steffen et al., 2002; Schäfer and Strimmer, 2005; Berger et al., 2007; Langfelder and Horvath, 2008; Parkkinen and Kaski, 2010; Weng et al., 2011) for the analysis of transcriptomes have been developed. A basic assumption made by many of these co-expression methods is that relationships in a biological network can be accurately described using linear dependence measures such as Pearson correlation or a monotonic dependence measure such as Spearman's correlation. However, linear or monotonic relationships approximate only a fraction of the true relationship types observed in a biological system (Figure 1). By limiting subsequent analysis to the linear fraction of the relationships in the biological network, researchers limit their ability to accurately recreate the network and identify the proper gene modules. One means of circumventing this problem has been through the use of Mutual Information (MI), which is capable of identifying non-linear connections in the data, and has been used in several previously described algorithms (Butte et al., 2000; Daub et al., 2004; Margolin et al., 2006; Meyer et al., 2007). A drawback of MI, which has proven difficult to address in some cases, has been its sensitivity to bin size and number as well as an unsatisfying [0-Infinity] range (Reshef et al., 2011). Recently, a modification to MI termed Maximal Information-based Non-parametric Exploration (MINE) has been described that eliminates these two limitations of MI by identifying the ideal bin size and renormalizing the MI measure into a [0,1] state space (Reshef et al., 2011). We utilize MINE in Maximal Information Component Analysis (MICA) to construct networks that are based on a more accurate set of relationships.

Another common assumption made by many module construction algorithms involves the method by which genes are clustered into modules after the underlying network structure has been identified. Many methods adopt a strict clustering approach, where genes are partitioned uniquely into a single module per gene. In some cases, this is done out of necessity (hierarchical tree-based methods), but in many cases it is done purely for computational efficiency. Although convenient and fast, clustering methods that force genes to uniquely exist in a single module result in incomplete modules, missing key genes that link the modules to one another (Parkkinen and Kaski, 2010). An alternate approach assigns “fuzzy” module membership (MM), in which genes can exist in multiple modules simultaneously (Yang, 1993; Daub et al., 2004; Yang et al., 2004; Parkkinen and Kaski, 2010). In MICA, we apply interaction component modeling for genes (ICMg), an iterative module identification method that assigns “fuzzy” MM based on the empirical results of the Latent Dirichlet Allocation algorithm (Parkkinen and Kaski, 2010). By not relying on traditional one to one gene-module approaches, we allow for a more accurate reconstruction of module dynamics and relationships to clinical traits of interest to the researcher.

In this paper, we describe a novel module identification method, MICA, which avoids some of the above unlikely assumptions made by other network algorithms. We then demonstrate its functionality over prior methods by analyzing two large gene expression datasets collected from macrophages and liver from about 100 inbred strains of mice.

## Results

We developed MICA as a means to address what we viewed as problematic assumptions made by many other network analysis algorithms. The method relies on the combination of two previously described methods, each of which addresses a one of our primary concerns with current methods. In order to account for the many non-linear interactions which we have observed in our data, we utilized a recently described algorithm, MINE, which can identify and measure both linear and non-linear interactions. We paired this with the ICMg algorithm, which utilizes the data generated by MINE to place genes into multiple modules, which accounts for the multiple interactions in different pathways that genes may have.

For this paper, we utilize two datasets, one on control and treated macrophages and the other from livers. Both datasets were taken from the Hybrid Mouse Diversity Panel (HMDP), a large mouse panel of over 100 strains of mice (Ghazalpour et al., 2012). Millions of SNPs and other genetic perturbations exist between the strains of mice in the HMDP while confounding factors such as environmental variation are minimized, making these datasets ideal for network biology and module identification. Both datasets were verified to have large enough sample sizes to reliably address issues of non-linearity. We first describe the components which make up the MICA algorithm, then compare the results of MICA to the well-regarded weighted gene co-expression network analysis (WGCNA) method (Langfelder and Horvath, 2008) on each dataset.

### MICA allows genes to exist within multiple modules

Gene modules attempt to represent groups of genes that act together in a concerted manner. The degree to which a gene belongs to a particular module, a measure known as MM, is a powerful tool for determining the relative importance of individual genes in a given module. In the context of WGCNA, the MM is defined as the correlation of a gene with the module representative (eigengene), and is sometimes also referred to as the module eigengene-based connectivity (kME) (Horvath and Dong, 2008).

**Figure 2:**
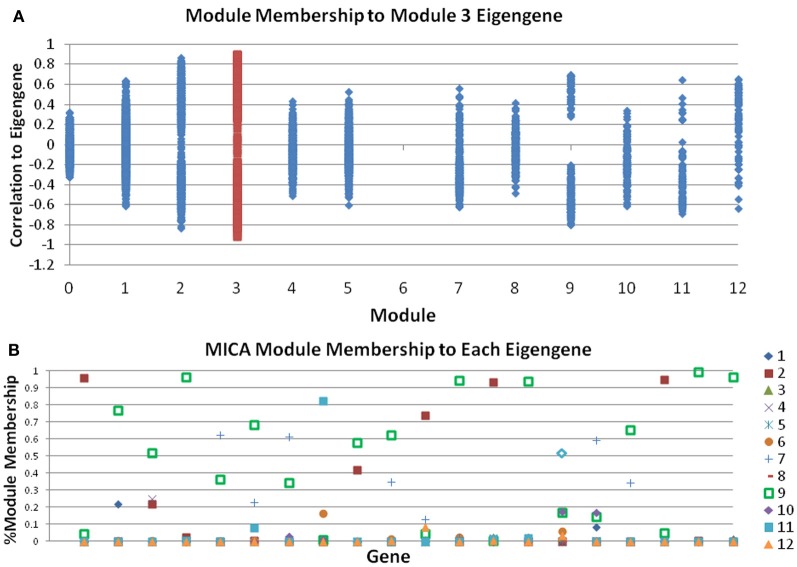
**Module Memberships are more meaningful in MICA. (A)** Module membership comparisons between all modules and the eigengene of module 3 for the macrophage dataset as calculated by WGCNA. Many genes in other modules, notably modules 2 and 9, have stronger correlations with the eigengene of module 3 than genes in module 3 itself. **(B)** Sample output from 20 genes from MICA, which calculates Module Membership before module analysis and the generation of the eigengene, meaning that all genes contribute to the calculation of each eigengene.

While many genes perform only a single role, and would be expected to reside in a single module (have high MM for one module, very low MM for all others), there are other genes that may play roles in multiple pathways. For instance, a transcription factor can activate multiple different pathways; Cytochrome C, which usually is responsible for energy metabolism, also plays an important role in the activation of apoptosis. These genes would have high MM in several modules corresponding to their important roles in each. Therefore, a critical step of network analysis is the calculation of the MM measure. However, by definition the MM measure in WGCNA and other methods are defined on already determined modules. As a result, this approach often produces confusing results. Genes that are placed in other modules will sometimes have higher MMs than many genes within a module. The genes with low MM within a module are counted fully, while those outside are ignored when summarizing the module in question (Figure 2A). MICA calculates MMs prior to actual module assignment, which allows all genes with high MM for a module to affect the module, while limiting the effects of genes with low MM. Figure 2B shows a sample set of 20 genes taken from our macrophage dataset after analysis with MICA. Several distinct patterns of gene expression can be observed, with most genes showing strong membership in a single module, while others appear to act across two or more modules, including several which do not appear to belong predominantly in any module. By using a weighted PCA algorithm, it is possible to fully incorporate the contributions of each gene to each module, regardless of the magnitude of that contribution.

### MICA reproduces scale-free topology

Work by Barabási and others has suggested that the underlying topology of biological networks is approximately “scale-free” (Langfelder and Horvath, 2008; Barabási et al., 2011; Dewey et al., 2011). In other words, the distribution of node connectivities approximates a power law distribution. Approximate scale-free topology (SFT) has been empirically observed in studies performed on metabolite networks and protein–protein interaction networks (Barabási et al., 2011). Several popular module construction algorithms, including WGCNA, evaluate the fit of their preliminary co-expression network against a SFT. These approaches then systematically modify their co-expression networks in order to maximize the goodness-of-fit to the *a priori* scale-free assumption prior to module partitioning. In the case of WGCNA, Zhang and Horvath (2005) observed that the scale-free fit of a correlation network is highly dependent on the significance threshold used for thresholding the correlation coefficient. They proposed the SFT criterion, which functions by raising each element of the correlation table to a series of powers and comparing the resulting correlation distributions to an idealized SFT distribution. Users are recommended to choose the smallest exponent that allows the scale-free goodness-of-fit criterion to surpass a given threshold (usually an *R*^2^ of 0.9). Raising the correlation matrix to a user-defined power in this way is a significant and severe modification to the original network relationships, with higher powers increasingly distorting the data to favor only the strongest possible connections while devaluing weaker connections.

**Figure 3:**
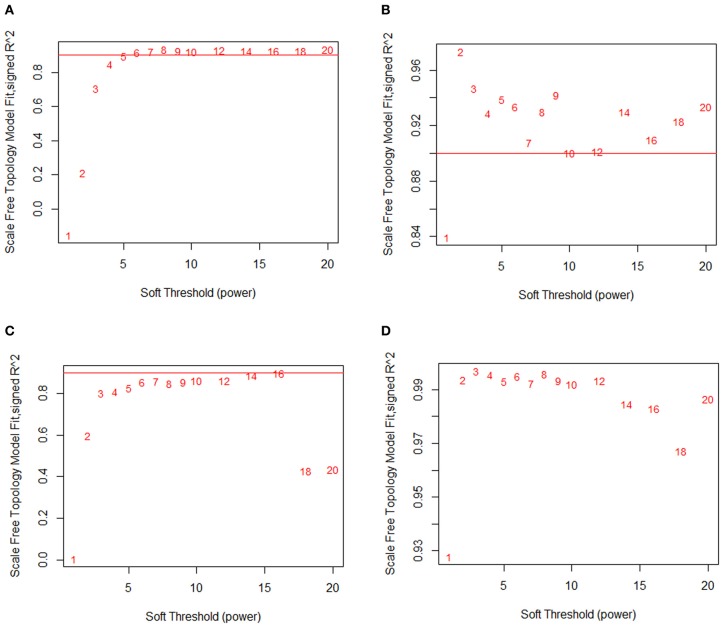
**MINE returns a scale-free network structure.** Scale-free topology fits at various powers of the relationship matrix. **(A)** Macrophage and Pearson correlation. **(B)** Macrophage and MINE. **(C)** Liver and Pearson correlation. **(D)** Liver and MINE. At a power of 1, Pearson correlation shows no indication of scale-free topology, while MINE shows strong evidence of scale-free topology.

When Pearson correlation is used for constructing a correlation network, the SFT criterion typically requires one to choose a relatively high power (6 or greater). Using Pearson correlation, we observed that for the macrophage dataset, SFT was only achieved after raising the correlation matrix to the power of 7 (Figure 3A). Likewise, the liver dataset requires a power of 16 (Figure 3C). At a power of 1 (the original correlation table without modification), the SFT score is negative for the macrophage data and very close to zero for the liver data, indicating a profound disagreement between the raw output of Pearson correlation and an acceptable SFT fit.

In comparison, Maximal Information is a modified version of MI that accurately identifies both linear and non-linear relationships. Strikingly, we find that very low powers are needed to achieve SFT when MINE is used both for the macrophage (Figure 3B) and liver (Figure 3D) data. We observe that MINE gives a nearly ideal fit to a scale-free network at a power of 2 for macrophage and already passes the recommended threshold at a power of 1 (unmodified) for the liver data. Without modification, the macrophage dataset nearly passes the threshold as well. This suggests that the MINE algorithm naturally captures the hypothesized approximate SFT of biological networks, and eliminates the need to explicitly soft threshold the data with a power function.

### Principle component analysis is conserved across a wide range of potential MM cutoffs

**Figure 4:**
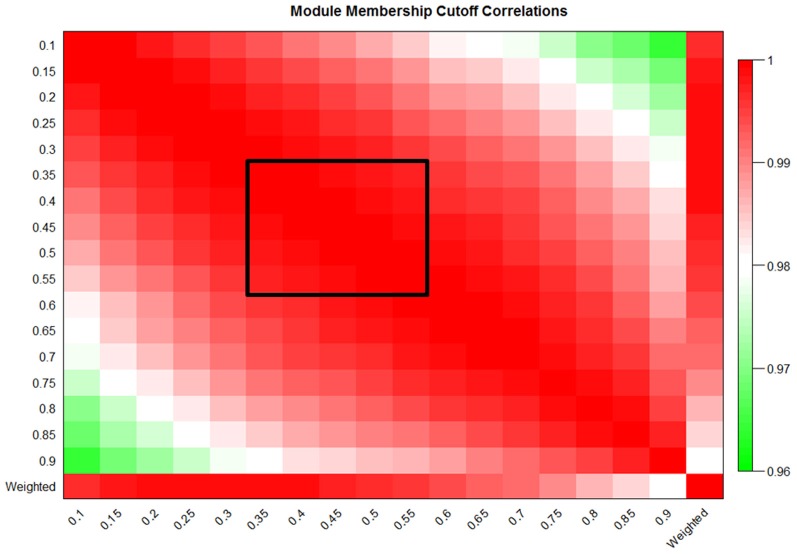
**Eigengene values are preserved over a large range of MM cutoffs in MICA.** Eigengene correlations at 5% cutoffs from 10% to 90% MM and also the weighted eigengene for the macrophage dataset. The black box indicates the region of preserved module eigengenes where correlation to one another and to the weighted PCA was greater than 0.99.

Two common goals of module construction algorithms are the identification of enriched pathways, domains, and molecular functions within modules, and the discovery of modules which are strongly correlated with disease severity or other phenotypes of interest (Sharma et al., 2005; Weng et al., 2011). Gene-set enrichment algorithms calculate the overabundance of a particular category of genes within a group when compared to that category's presence in the entire dataset (Huang et al., 2009a,b). To calculate overrepresentation, these methods require strict binary categorization of genes as either being present or absent from a given module. Using MICA, any MM cutoff could theoretically be selected to perform this categorization; however, proper MM cutoffs should preserve the overall action of the MICA-identified module. In order to determine the stability of the network at various MM cutoffs, we calculated the first principle component [called an eigengene (Park et al., 2011)] of each module in our MICA-derived macrophage network at seventeen MM cutoffs (5% intervals from 10% to 90% MM). We then calculated the average correlation of eigengenes to one another and to the weighted PCA which represents the true activity of the module as a whole without partitioning. This stability measurement was high across the panel of MM cutoffs, with a significant loss occurring only when MM cutoffs were greater than 70% or less than 20%. Between 35% and 55% MM cutoffs, eigengene correlations to one another and to the weighted PCA were greater than 0.99 (Figure 4). This near perfect correlation implies that within this range of cutoffs, any binary partitioning of the modules is equally capable of describing the action of the network as a whole.

### Stability of eigengenes allows for selection of optimal modules in terms of size and gene-set enrichment

**Figure 5:**
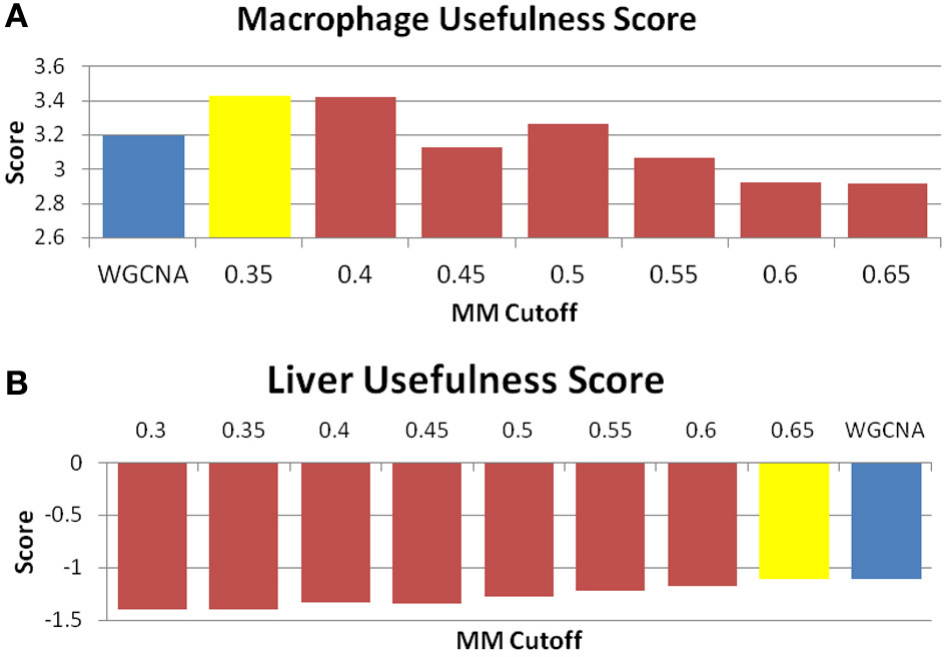
**MICA displays equal or higher “usefulness” than WGCNA.** “Usefulness” score of data for the **(A)** macrophage and **(B)** liver datasets. The blue bar is the score received by WGCNA, while the yellow bar is the score returned for MICA at the optimal MM cutoff. A higher score indicates a more desirable result. We observe an improvement in the macrophage and conservation in the liver datasets.

Ideally, network analysis and module construction should prioritize specific pathways and genes for further analysis by targeted approaches. To achieve that goal, ideal modules should be both highly enriched for specific gene categories, and also small enough to reasonably examine all the genes in the module for interesting candidates and drivers without eliminating large numbers of genes from consideration. In MICA, average module size is inversely correlated with MM cutoff, but the relationship between MM cutoff and gene-set enrichment is significantly more complex. We observe near perfect correlation (greater than 0.99) in the MICA modules for cutoffs that lie between 35% and 55%. This implies that we may select any cutoff within this range for gene enrichment analysis and remain confident that the modules selected accurately represent the entire network as a whole. To determine this ideal cutoff and identify the optimal modules for further analysis, we calculated DAVID enrichment scores for each set of modules (Dennis et al., 2003; Huang et al., 2009b). We then applied a metric that incorporates both module sizes and enrichment significances while penalizing the network for the number of genes not included in the overall network in order to determine the optimal MM cutoff to use for further analysis (Figure 5A). For example, in the macrophage dataset, the optimal MM cutoff is 35% a point where the binary partitioned model represents the network as a whole and possesses several small but highly enriched modules.

### Comparison of MICA to WGCNA

WGCNA is an extensively used module identification and network analysis method (Zhang and Horvath, 2005; Langfelder and Horvath, 2008; Dewey et al., 2011; Park et al., 2011). We compared the MICA method to WGCNA using two recently described gene expression microarray datasets from a large mouse panel, one from control and OxPAPC-treated macrophages (Orozco et al., 2012) and another from liver (Bennett et al., 2010). We constructed modules in WGCNA using the standard methodology described in Langfelder et al. (2008). WGCNA infers the number of modules in a co-expression network automatically based on dynamic branch cutting of a hierarchical cluster tree (Langfelder et al., 2008). Additional modules can create bias due to additional degrees of freedom. In order to prevent bias, we fixed the number of modules for MICA to the same number that was inferred through WGCNA.

In comparing WGCNA to MICA, we rely on several measures of network fitness. The first measure of network fitness is the SFT criterion defined by comparing the observed distribution of edge connections across the inferred network to the power-law distribution of an ideal scale-free system. WGNCA suggests raising the correlation matrix to a power in order to reach an appropriate approximation to SFT. We use this method when comparing MICA to WGCNA, observing at which power each method reaches an appropriate approximation to a scale-free system. A method that better captures the SFT of the underlying network is the one that reaches this scale-free criterion threshold at the power closest to unity. The next comparison metric is perplexity, a measure of the entropy of a system, and equivalent to a misclassification rate (Brown et al., 1992; Parkkinen and Kaski, 2010). We constructed standard gene classes as described in Shiga et al. (2007) and calculated the ability of either WGCNA or MICA to recapture these classes in their modules. Network analysis methods are often used, particularly in datasets which only vary due to biological variability to determine Gene Ontology (GO) categories for further study (Gargalovic et al., 2006; Horvath et al., 2006; Dewey et al., 2011; Yee et al., 2011; Xiao et al., 2012). We utilized differences in GO enrichments as one measure of network fitness, but felt that a strict comparison of GO enrichment values only captured part of the overall “usefulness” of the constructed modules. To address this issue, the final measure of network fitness compares modules identified through MICA and WGCNA by their “usefulness” as determined by a combination of DAVID gene-set enrichment, module size and number of genes unplaced in modules (Huang et al., 2009b). Ideally, as many modules as possible in a network should be highly enriched and reasonably small to assist in further study.

### Macrophage dataset

We examined a dataset consisting of the 5070 most variably expressed genes in a panel of macrophages isolated from inbred mouse strains before and after treatment with OxPAPC, an oxidized phospholipid. MICA strongly captures the SFT in the system, crossing the recommended threshold at a power of 2 and attaining a nearly perfect fit to an ideal scale-free system with an *R*^2^ of 0.97 (Figure 2B). At a power of 1 (the raw relationship values), the scale-free fit is very high at 0.84. By comparison, the Pearson correlation used by WGCNA does not reach the scale-free threshold until a power of 6 (Figure 2A). At the power of 1, it is clear that the Pearson correlation is not an accurate means by which one may capture the SFT of this system, as the signed *R*^2^ value of the degree distributions is less than 0.

**Figure 6:**
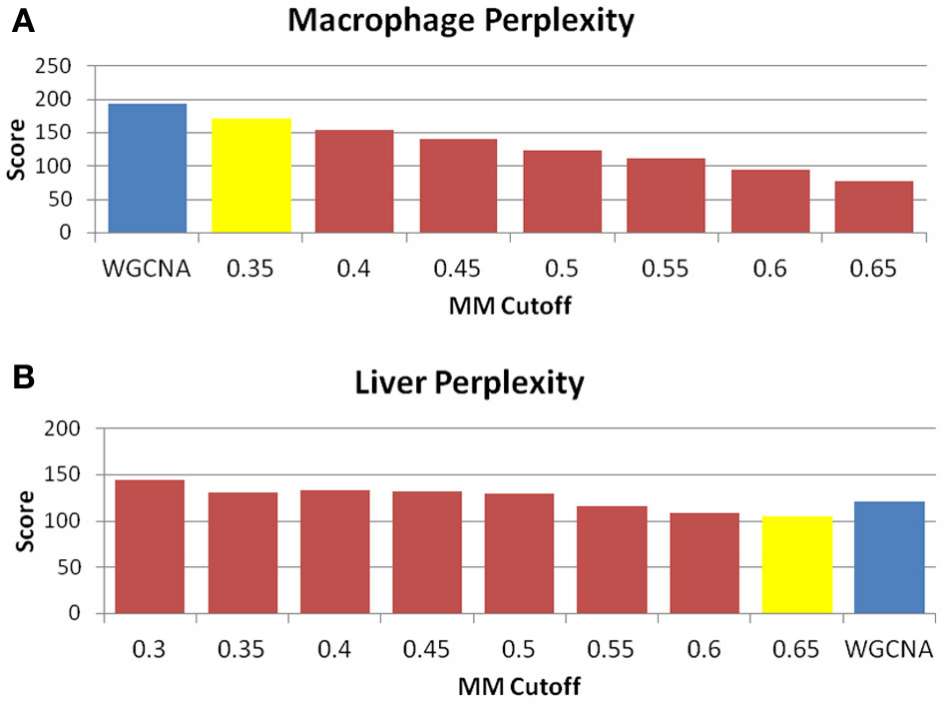
**MICA returns a small improvement in module entropy over WGCNA.** Perplexity measures for **(A)** macrophage and **(B)** liver. The blue bar is the score received by WGCNA, while the yellow bar is the score returned for MICA at the optimal MM cutoff. As perplexity is a measure of entropy, a lower score is more desirable. In both cases, a small improvement in perplexity is observed in the optimal MICA modules vs. the WGCNA modules.

**Figure 7:**
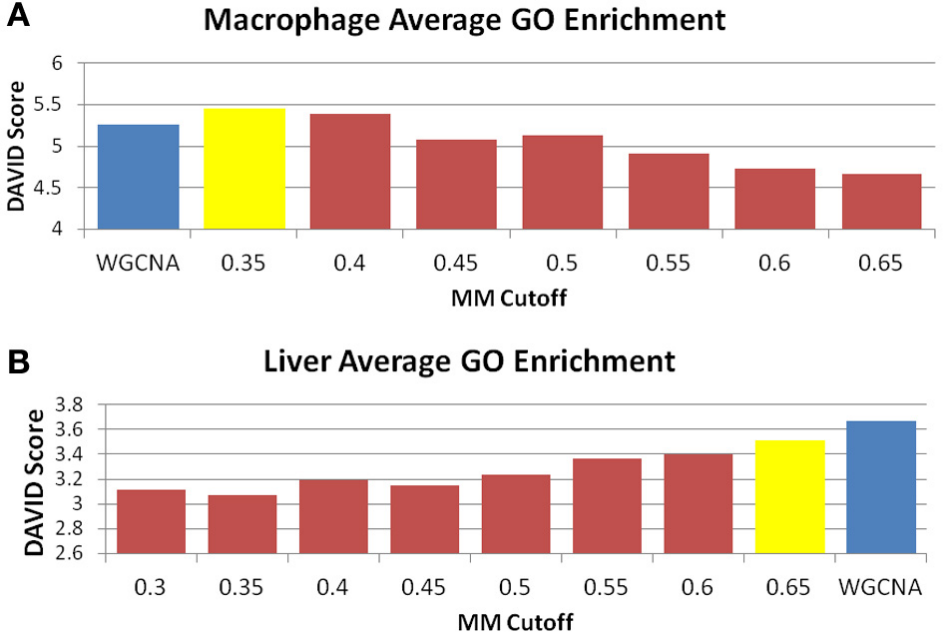
**MICA has higher average GO enrichment in the macrophage dataset.** Average GO enrichments for modules derived from **(A)** macrophage and **(B)** liver datasets. The blue bar is the score received by WGCNA, while the yellow bar is the score returned for MICA at the optimal MM cutoff. A higher score is more desirable, and we observe improved average GO enrichment for MICA in the macrophage data, and improved average GO enrichment for WGCNA in the liver data.

The perplexity of the MICA-derived modules varied significantly based on the MM cutoff selected. In comparing WGCNA and MICA, we chose to compare WGCNA to the ideal MM cutoff selected by our “usefulness” measure, which combines gene enrichment and module sizes. WGCNA returned a perplexity score of 193.44 based on 256 standard GO categories included in the analysis (Figure 6A). The ideal MM cutoff for the macrophage dataset is 35%. At that cutoff, the MICA modules have a perplexity score of 171.77, a 11.2% improvement over WGCNA. We also calculated perplexities at 5% intervals across the stable range of MICA (35–60%) (Figure 6A). In terms of our module “usefulness” measure, we saw improvement (3.43 vs. 3.20) between the 35% MM cutoff MICA modules and the WGCNA modules. Comparable improvement was observed at 40%, while equivalent enrichment was observed at 50%. (Figure 5A). Also observed were increases in average GO enrichment at 35 and 40% cutoffs compared to WGCNA (5.457 and 5.384 vs. 5.264, Figure 7A).

Orozco et al. (2012) describe a set of genes and GO terms which are involved in the OxPAPC response. In order to determine the ability of MICA and WGCNA to return relevant modules, we examined each set of modules and compared them to the results of Orozco et al. Terms of interest included regulation of kinase activity, cytokine production, genes containing a SH2 domain, glutathione biosynthesis, and oxidative stress response. We compiled lists of all enriched GO terms in both WGCNA and MICA modules. We observed that the MICA-analyzed network contained more modules that were significantly enriched for these GO terms, with six modules being enriched for one or more term of interest as opposed to four in WGCNA. Both methods were able to identify modules involved in oxidative response, regulation of kinase activity and cytokine production, while WGCNA identified an additional module involved in glutathione metabolism and MICA identified two modules associated with SH2 domain and an additional module for regulation of kinase activity. We also observed that MICA segregated all identified OxPAPC-related genes (Hmox1, Ifi205, and Il1a) into a single module, while WGCNA split these genes into multiple modules. The identification of a core “OxPAPC response” module, as defined as the module which contains all the OxPAPC-related genes, represents a significant improvement for MICA over WGCNA, which was unable to find such a module.

### Liver dataset

We also examined a dataset consisting of 7000 highly expressed genes from livers taken from a large panel of mouse strains. In these data we observed MICA strongly capturing the SFT of the network, reaching an *R*^2^ fit of 0.93 without any modification and an *R*^2^ greater than 0.99 at a power of 2 (Figure 3D). By comparison, Pearson correlation did not reach the recommended cutoff of *R*^2^ = 0.9 until a power of 16, representing a substantial modification of the co-expression data in order to fit the underlying hypothesis (Figure 3C). As in the macrophage data, the unmodified Pearson correlation data showed no relationship to a scale-free network, with an *R*^2^ close to 0.

**Figure A1:**
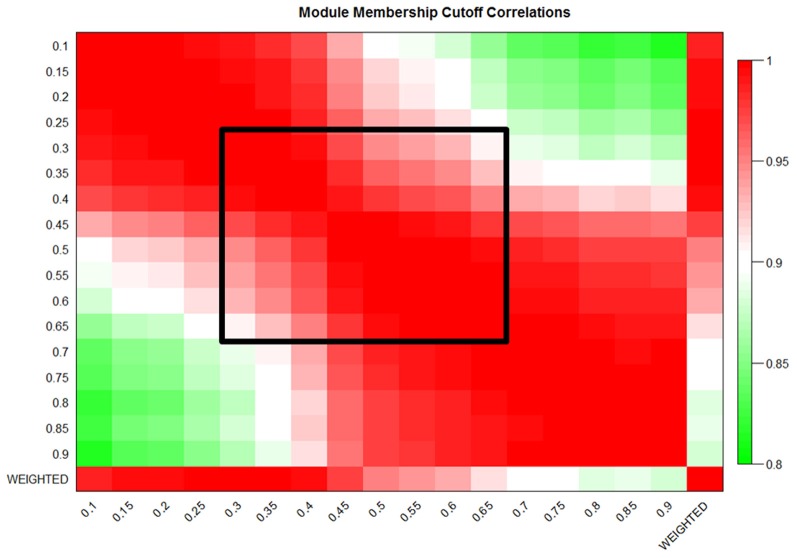
**MICA shows strong preservation of eigengenes in the liver dataset.** Eigengene correlations at 5% cutoffs from 10% to 90% MM and also the weighted eigengene for the liver dataset. The black box indicates the region of preserved module eigengenes.

Unlike the macrophage dataset, we do not observe the same level of conservation of eigengenes across MM cutoffs in the liver dataset. We selected for further analysis a set of MM cutoffs (35–65%) in which eigengene correlations were over 0.9 (Figure A1). We also observe that the GO enrichment terms generally improve rather than decrease over the range of conserved MM cutoffs, and our ideal MM cutoff occurs at 65%. At 65%, MICA returns a perplexity score of 105.38, while WGCNA returns a perplexity score of 121.02 (Figure 6B). MICA shows a 12.9% improvement over WGCNA in terms of perplexity for the liver dataset.

WGNCA was unable to place 66.5% of genes into modules, which affected its “usefulness” score compared to MICA, which was unable to place 10.9%. However, WGCNA returned higher average GO enrichments (3.67 vs. 3.51, Figure 7B) when compared to MICA. Modules were indistinguishable from one another in terms of overall module usefulness (−1.10201 vs. −1.10213, Figure 5B).

### Module stability

In order to determine the overall stability of the modules observed in both WGCNA and MICA, we randomly partitioned the macrophage dataset into two equal parts and ran both MICA and WGCNA on each half. No universally accepted means of comparing two sets of modules to one another exists, particularly in the case of modules with non-binary gene-module occurrence. We adopt a method previously used to compare modules created by WGCNA (Langfelder and Horvath, 2008) to compare the MICA and WGCNA modules to one another. We note that this method was designed for network methods which place genes into single modules, and that forcing our MICA results to conform to this requirement will inevitably weaken the network stability observed via MICA.

We observed that when run through the soft thresholding function, both MICA runs return a power of 3, while the two runs of WGCNA differ, with one returning 4 and the other 6. This suggests that MICA is capturing similar levels of SFT for each portion of the data while WGCNA is unable to do so. The hard thresholding criteria for both MICA runs is also identical at a cutoff of 0.45. We further observe that WGCNA returns differing numbers of modules (13 vs. 14) for the two halves of the macrophage dataset.

**Figure 8:**
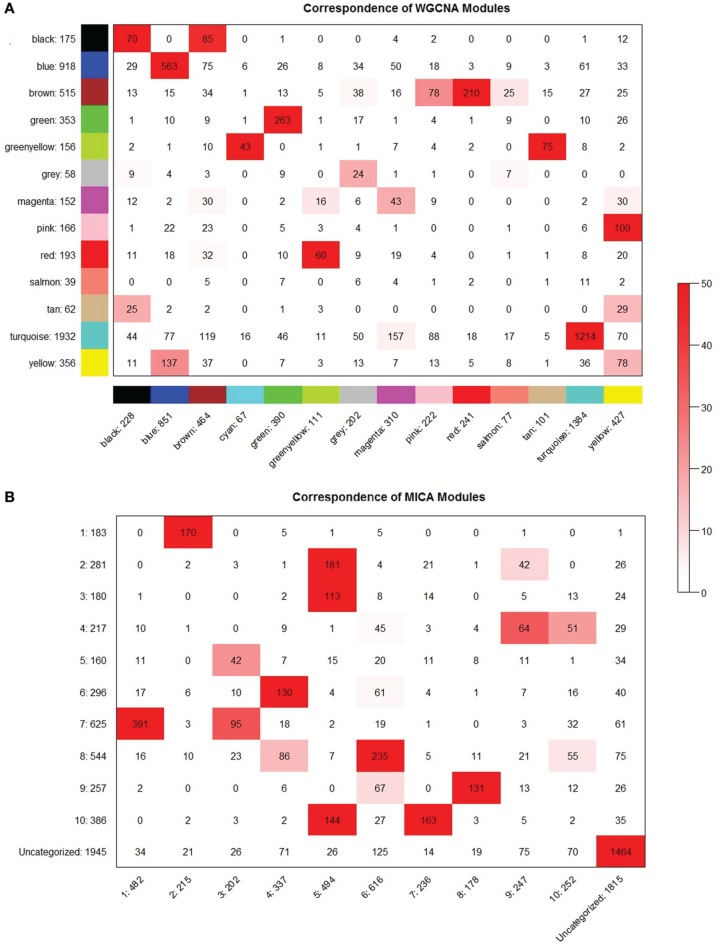
**MICA and WGCNA have comparable module preservation statistics.** Module preservation between even partitionings of the macrophage dataset for **(A)** WGCNA and **(B)** MICA. Preservation significances are calculated using a Fisher's exact test and are represented by the depth of the red color as indicated.

We observe broadly similar levels of stability in both WGCNA- and MICA-derived modules (Figure 8), with the majority of modules in both methods showing strong preservation between the halves of the macrophage dataset. A notable exception is the salmon module from part 1 of the WGCNA data (Figure 8A), which is not preserved at all in the part 2 WGCNA network. The salmon module of part 2 of WGCNA also shows relatively weak preservation as well.

### Effects of dataset on MICA

We find that MICA appears to show an overall improvement in module construction when compared to WGCNA in the macrophage dataset, but is comparable to WGCNA in the liver dataset. To evaluate whether underlying differences in the network architecture between the two datasets led to the differences in improvement, we returned to the original data to look for differences in the number of non-linear interactions captured by MINE vs. Pearson correlation. If there are more non-linear interactions in a dataset, then MICA should perform better than WGCNA, which does not take into account the non-linear interactions in the data. On the other hand, if a network has very few non-linear interactions, then both MINE and Pearson correlation should return comparable results to one another.

**Figure 9:**
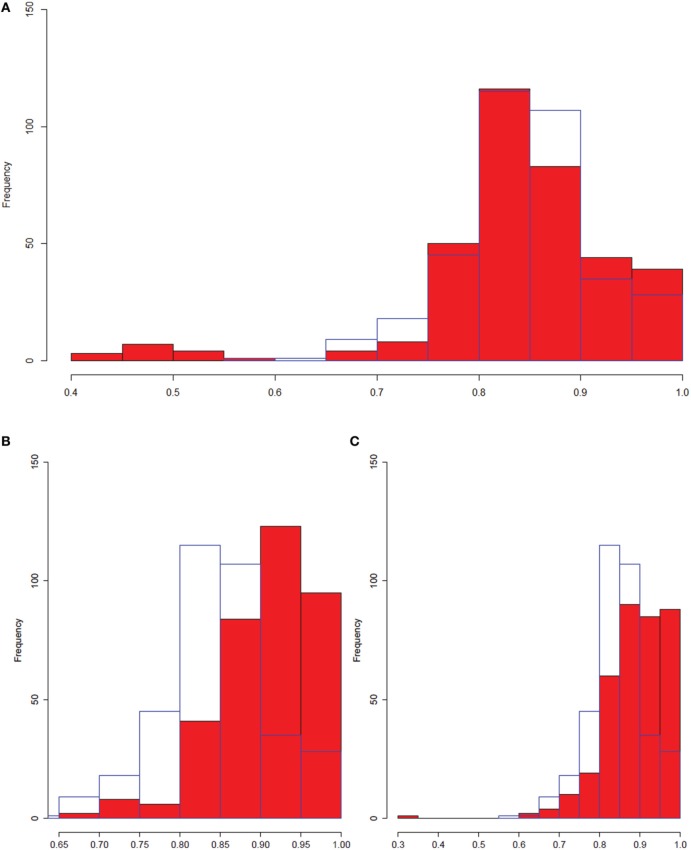
**The entire macrophage dataset has a greater number of non-linear interactions.** Histograms comparing distributions of Pearson correlations for interactions with high (>0.9) MIC scores. All distributions were sampled such that there were 360 entries for each entry. The blue border in each part of the figure is the liver dataset. **(A)** Full macrophage dataset vs. liver dataset. **(B)** Control macrophage vs. liver. **(C)** Treated macrophage vs. liver. We observe an enrichment for low Pearson correlation data with high MIC scores (implying significant non-linearity) in the full macrophage set which is not observed in either the control, treated, or liver datasets.

In order to determine whether we were seeing more non-linear interactions in the macrophage dataset, we selected all relationships from both the macrophage and liver datasets that had a high (greater than 0.9) maximal information coefficient (MIC) score. Our first observation was that the macrophage dataset had significantly more strong MIC scores than the liver dataset (1274 vs. 360 interactions). We then examined the distribution of the Pearson correlation values measured for these strong MIC interactions, after sampling the macrophage dataset such that it had an equal number of observations as the liver data (Figure 9A). Compared to the liver data, the macrophage data showed enrichment for both very high (greater than 0.9) and low (less than 0.6) Pearson correlations. This suggests that the macrophage dataset both contains more non-linear interactions, and also a greater fraction of interactions that are very close to perfectly linear. While the linear interactions will be picked up by Pearson correlation, the increased number of non-linear interactions can only be detected appropriately through MINE.

There are two possible explanations for the differences between the two datasets. The first is that the macrophage dataset is an *in vitro* system containing a single cell type, while the liver samples contain multiple cell types. The second possible explanation is that the improvement comes because we analyzed both treated and untreated data together, rather than separately. Accordingly, we separated the control and OxPAPC-treated macrophages and compared each separately to the liver dataset (Figures 9B,C). We observed slightly increased numbers of strong MIC interactions for the control (448) and treated (549) data compared to the liver data (360). However, although we continued to observe enrichment of very high correlations in both the control and treated OxPAPC datasets compared to the liver data, and we no longer observed enrichment of low correlations in either data (with the exception of a single interaction in the OxPAPC-treated dataset). This is an example of gene by environment interactions where a treatment or environmental perturbation interacts with underlying genetic variation to result in different relationships between genes in different environmental conditions. These observations suggest that the improvement observed when using MICA on the macrophage dataset is a result of MICA's ability to capture gene by environment interactions between the treated and control samples. It further suggests that Pearson correlation and WGCNA are less successful in the macrophage dataset because they are incapable of using these interactions.

## Discussion

We report a novel network analysis method, MICA, which combines two previously published methods: MINE, a modification of MI which accounts for non-linear interactions in datasets without many of the shortcomings of the canonical methods, and ICMg, which relies on an iterative process to assign distributed MMs as opposed to a rigid in-or-out dichotomy. Together, this combination is less restrictive than module construction algorithms that include linear but exclude non-linear co-expression relationships and allow only single-MM. Thus, MICA has the advantage that it embraces concepts that are better rooted in actual biological observations.

To validate the MICA approach, we analyzed datasets from macrophages treated with OxPAPC and from livers, which in one case revealed distinct advantages of MICA over WGNCA, a benchmark correlation network approach, and yielded comparable results in the other case. Specifically, MICA may be particularly well suited for the analysis of networks in which gene by environment interactions are expected to occur, which traditional module construction methods are ill-equipped to detect. In the case of macrophages treated with OxPAPC, analysis with MICA resulted in modules that are more highly enriched in pathways of interest, and better able to place genes with similar functions into the same modules compared to other methods. In both macrophage and liver datasets, there is a dramatic improvement in the ability for MICA to detect an overall network structure that better approximates the hypothesized topology underlying the biological network.

**Figure 10:**
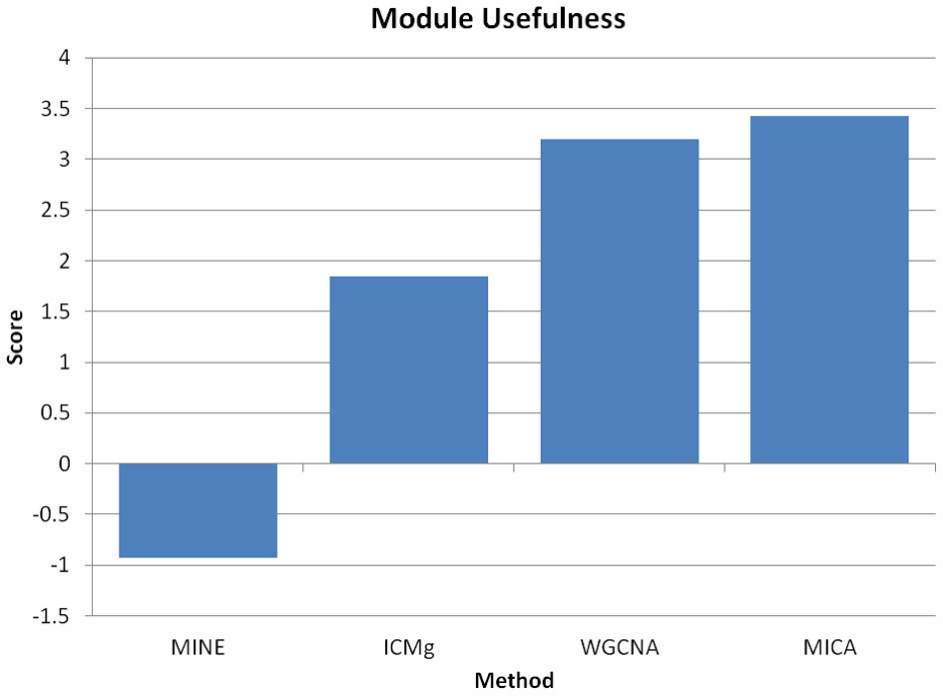
**MINE or ICMg used separately do not improve network analysis.** Module “usefulness” measure for macrophage data when using either MINE plus hierarchical clustering, Pearson correlation plus ICMg, WGCNA, or MICA. Higher usefulness is more desirable, and we observe significantly reduced usefulness scores when either MINE or ICMg are used separately from one another.

We have further observed that in contrast to MICA, which utilizes MINE and ICMg, no significant improvements were achieved when WGCNA was modified by using ICMg on Pearson correlations, or topological overlap and hierarchical clustering on Maximal Information scores (Figure 10). Thus, both MINE and ICMg each provide partial solutions that are synergistic when combined.

As an attempt to incorporate known biological principles such as feedback loops and multi-functional proteins into a transcriptome co-expression network analysis method, MICA shows initial promise, but will undoubtedly benefit from further refinement. A major drawback is that MICA lacks the ability to independently determine the number of modules in a network; i.e., an arbitrary number of modules must be specified. Additionally, it is not straightforward to calculate *p*-values (significance) for the MICA measure, in contrast to the many approaches that have been developed (e.g., regression models) to calculate *p*-values for correlation measures. Finally, MICA is significantly more computationally intensive than correlation-based methods such as WGCNA. Such improvements may allow MICA to identify smaller, more enriched and more relevant modules for further analysis and discovery of novel genes with roles in important phenotypes.

In conclusion, MICA is an attractive network analysis method because (1) it does not discard non-linear interactions; (2) it removes the need for soft thresholding; (3) employs a fuzzy clustering algorithm for module detection; and (4) shows improvements over correlation algorithms in certain cases, particularly those involving gene by environment interactions.

## Methods

### Maximal information non-parametric exploration (MINE)

We utilize the recently described MINE algorithm to determine a normalized relationship matrix which incorporates non-linear interactions (Reshef et al., 2011). MINE relies on a modified version of MI called the MIC. MIC calculates normalized MI values for all partitions of a finite set of ordered pairs with the *x*-values going into x bins and the *y*-values partitioned into y bins, such that *x* × *y* < *n*^0.6^, as recommended by the authors and where n is the number of arrays. The algorithm then normalizes across partitions with the same number of bins, but different bin sizes, by dividing the data by log(min{*x*, *y*}), which is the maximum possible score for any MI query with x horizontal and y vertical bins. The MIC of an interaction is then defined as the maximum normalized value across the set of partitions. MINE is implemented in a Java environment. MIC scores are calculated for all pairs of expression data and compiled into a matrix format.

### Scale-free topology

The soft thresholding SFT function of the WGCNA R package (Langfelder and Horvath, 2008) was used to determine the fit of all datasets and relationship generating methods (either Pearson correlation or MIC) to an idealized SFT. Briefly, the function acts by calculating the sum of the link strengths for each gene in the data, and finds an *R*^2^ between the distribution of total node link strengths and a power-law distribution. It then repeats this process, raising the original relationships to a power of *n* = 1 – 20. The ideal soft thresholding criterion is defined as the first power which passes the recommended *R*^2^ threshold of 0.9.

### ICMg

ICMg (Parkkinen and Kaski, 2010) relies on an iterative component model to calculate MMs. As ICMg does not allow for weighted edges, network edges were trimmed using the hard thresholding function of the WGCNA R package, which calculates an *R*^2^ fit between the degree of node connectivity in a dataset based on a thresholding function at increasing intervals of 0.05 and a power-law distribution. The ideal hard thresholding criterion is the lowest cutoff which passes a recommended *R*^2^ threshold of 0.9. ICMg allows users to select an arbitrary number of modules. As WGCNA automatically selects the number of modules it will return, we selected a number of modules equal to that observed with WGNCA for ICMg in order to avoid biasing the results toward the method with more modules and therefore more degrees of freedom. Module assignments were then initially assigned to the network using a Dirichlet distribution.

ICMg is an iterative process. In each iteration, each edge is independently interrogated utilizing Gibbs sampling with the following equation:

$$p\text{​}\left(z_{0}|{\left\{z\right\}}^{\prime },{\left\{L\right\}}^{\prime },\alpha ,\beta \right)\propto \frac{n_{z_{0}\;+\;\alpha }^{\prime }}{N^{\prime }+C_{\alpha }}\times \frac{\left(q_{z_{0}i_{0}}^{\prime }+\beta )(q_{z_{0}j_{0}}^{\prime }+\beta \right)}{\left(2n_{z_{0}}^{\prime }+1+M\beta )(2n_{z_{0}}^{\prime }+M\beta \right)}$$

where {*L*}′ is the set of all links excluding the one being interrogated, {*z*}′ is the set of module assignments for the links excluding the link being interrogated, *n*_*z*_ is the count of links assigned to component *z, i*, and *j* represent the genes linked by edge *z*_0_ and *q*_*z*_*i*__ counts the module-node co-occurrences between module *z* and node *i*. *C* is the total number of modules, and *M* is the total number of nodes. α and β are control parameters which modify the overall distribution of module sizes and the average MM per gene per module, however, these were not modified and the default values (α = 10, β = 0.1) found in (Parkkinen and Kaski, 2010) were used. 40,000 burn-in rounds were performed to eliminate any dependence on initial conditions and to allow values of *q/M* to reach steady-state values at which point MM of each node was sampled every 10 iterations for another 10,000 iterations of the network to determine proportional MM in each module for each gene.

### Determining the ability of MICA to address non-linearity in datasets

In order to determine whether the sample sizes of the macrophage and liver datasets were large enough to reliably address non-linearity, we utilized pre-computed bootstrapped tables of *p*-values for arrays of varying sample sizes from MINE available at http://www.exploredata.net/Downloads/P-Value-Tables. We observe that at our hard thresholding cutoff of 0.45, this means that for all edges in the liver MICA network, *p*-values are less than 2.72E-6, while for the macrophage dataset, all edges have *p*-values less than 2.74E-7, far over the nominal significance value of 0.05 or the Bonferroni corrected values of 7.1E-6 and 9.8E-6.

### Calculating “eigengenes” via weighted principle component analysis

We borrow the concept of the eigengene from WGCNA (Langfelder and Horvath, 2008) to describe the overall behavior of a set of genes. As in WGCNA, we define an eigengene of a module to be the first principal component of the transcript levels of the genes contained within the module, however, we utilize the dudi.pca function of the ade4 R package to implement a weighted PCA which utilizes the MMs from ICMg to weight the contribution of each gene to the eigengene (Chessel et al., 2004). We also calculate the unweighted eigengene for each module at 5% intervals across the genome in which genes whose MM for that module passes the current threshold are included in the eigengene calculation.

### Optimal MM cutoff selection

While there are methods to compare networks to one another (Langfelder et al., 2011), these typically are concerned with determining preservation of modules and comparing individual genes to one another and not asking which module is objectively “better.” In order to compare MICA networks to one another and to WGCNA-derived networks, we define a parameter “usefulness” (U), which incorporates both GO enrichment scores, the number of genes present in a given module and the number of genes not placed in any module. We define “usefulness” as follows:

$$U=\sum_{i}^{1,\ldots ,n}\left(\frac{{\text{DAVID}}_{i}}{\log_{2}N_{i}}\right)-\log_{10}M,$$

where DAVID_*i*_ is the maximum DAVID (Dennis et al., 2003; Huang et al., 2009b) enrichment score for module *i* (equivalently, the negative log of a GO enrichment score could be used), *N*_*i*_ is the number of genes in module *i*, and *M* is the number of genes not included in any module for the current method.

### WGCNA

We followed the network analysis methods described in Langfelder et al. (Langfelder and Horvath, 2008) and the parameters found in the online WGCNA tutorials at http://labs.genetics.ucla.edu/horvath/htdocs/CoexpressionNetwork/Rpackages/WGCNA/Tutorials/. Pearson correlations were determined for each pair of genes, and after performing a soft thresholding SFT fit, the correlations were raised to the recommended power. Adjusted correlations are then converted into Topological Overlap measures by the following equation:

$${\text{TOM}}_{ij}=\frac{\sum_{u}\left\{A_{iu}A_{uj}\right\}+A_{ij}}{\text{min}(k_{i},k_{j})+1-A_{ij}}$$

where *i* and *j* are the pair of genes to be analyzed, *u* is the set of all other genes, *A* is the adjusted correlation matrix, and *k* is the degree of the node. TOM scores are then converted to DistTOM scores by subtracting TOM from 1. The DistTOM array undergoes hierarchical clustering, and modules are determined using the dynamic tree cut algorithm and eigengenes are determined from the first principle component of the genes in each module. Modules whose eigengenes have a Pearson correlation of greater than 0.8 are merged.

The WGCNA method is implemented in the freely available WGCNA R package (Langfelder and Horvath, 2008). Here we used many of the R functions from this package (e.g., for evaluating SFT and the creation of Figures 4, A1).

### Standard gene classes

The GO database is organized into three distinct directed acyclic graphs. We derived standard gene classes for our data in a method similar to Shiga et al. (2007). Starting at the root of the Biological Process GO graph, we proceeded from parent node to child nodes, checking the number of genes in that GO category that also appear in any module in our gene networks. As we progress away from the root, the number of genes in each category decreases and the number of categories increases. We used the parameters utilized in Shiga et al. for our analysis. When a GO category contains less than 30 genes present in our network, we stop progressing down that branch and add its parent GO category to the standard gene-set, unless there are more than 300 included genes in that category, in which case it was omitted as being too broad for logical compartmentalization into a single module. In this way, we generate a set of reasonably sized functionally-related gene-sets with which to explore the accuracy of the module construction method using perplexity.

### Perplexity

Perplexity represents a measure of the entropy in a system, and has been used extensively in fields as diverse as natural language processing (Brown et al., 1992) to previous clustering algorithms (Parkkinen and Kaski, 2010). In this case perplexity represents the ability of a module creation algorithm to accurately recover underlying functional gene categories as determined by our standard gene classes. We applied perplexity to the confusion matrix formed of the frequency of co-occurrence between standard classes on the columns (*c*) and the modules as the rows (*r*). From this confusion matrix, perplexity is defined as

$$\text{perplexity}=2^{-\frac{\sum_{l}\text{log}\hat{P}(c_{l}|r_{l})}{N}}$$

where *N* is the total number of non-zero samples, *l* is an indexing variable for all such entries in the confusion matrix, and the probabilities $\hat{p}$ are empirically determined by normalizing the rows of the confusion matrix. Perplexity is proportional to the size of the overall dataset. To compare perplexities between network methods, we normalize the data by multiplying each perplexity value by the proportion of genes initially included in the dataset and the genes actually placed by each method. A lower perplexity score represents a more accurate capture of the functional categories.

### Module stability

Module stability was calculated using the method described by (Langfelder and Horvath, 2008) and documented at http://labs.genetics.ucla.edu/horvath/htdocs/CoexpressionNetwork/Rpackages/WGCNA/Tutorials/Consensus-RelateToFemMods.pdf. Briefly, the macrophage dataset was randomly divided into two halves. Each half was independently processed using MICA and WGCNA. MICA genes were forced into the module in which it had the highest MM to allow for the use of the method. In order to determine module preservation, each half was compared to one another by creating a table of gene-overlaps between genes in modules of the first half and genes in modules of the first half. A Fisher's exact test was applied to each overlap to calculate a significance of preservation for each module–module pairing. Overall module preservation was then visually determined based on the significance of preservation for each module in the other half of the dataset.

### MICA

Code for MICA is available from systems.genetics.ucla.edu

### Datasets

The macrophage dataset was obtained from Orozco et al, which isolated primary macrophages from a large panel of inbred mouse strains (Orozco et al., 2012). The macrophage dataset includes 80 strains of control macrophages and macrophages treated with 50 ug/ml oxidized 1-palmitoyl-2-arachidonoyl-sn-glycero-3-phosphatidylcholine (OxPAPC) for 4 h.

The liver dataset was taken from Bennett et al. and includes livers taken from 97 strains of mice (Bennett et al., 2010). Transcriptome data was obtained using the Affymetrix HT MOE-430A microarray platform, and normalized using the robust multichip average (RMA) method.

A major limitation of MICA is the time involved in the generation of the MIC scores using MINE, which has a large O(*n*^2^) computation time. In order to run MICA in a reasonable amount of time, it is important to limit the genes selected to the smallest informative set. As such, we selected for genes which were expressed in the dataset and which showed variation across the dataset (as genes which do not vary are generally uninformative for network analysis). We calculated average signal intensity and coefficient of variation (CV) for each probeset. We then reduced our dataset to relevant genes by first selecting probes with above average intensity, and then selecting probes with greater than 5% CV, resulting in 5070 genes for the Macrophage dataset. For the liver dataset, we selected the 7000 most highly expressed genes for analysis.

Both datasets are available at http://systems.genetics.ucla.edu/data/

### Conflict of interest statement

The authors declare that the research was conducted in the absence of any commercial or financial relationships that could be construed as a potential conflict of interest.
